# Protective Effect of Vitamins C and E on Depot-Medroxyprogesterone Acetate-Induced Ovarian Oxidative Stress In Vivo

**DOI:** 10.1155/2016/3134105

**Published:** 2016-02-07

**Authors:** Atik Ismiyati, I. Wayan Arsana Wiyasa, Dwi Yuni Nur Hidayati

**Affiliations:** ^1^Yogyakarta Midwifery Academy, Jalan Parangtritis Km 6, Sewon, Bantul, Yogyakarta Special Region 55188, Indonesia; ^2^Division of Fertility, Endocrinology and Reproduction, Obstetric and Ginaecology Laboratory, Saiful Anwar General Hospital, Faculty of Medicine, Brawijaya University, Malang, East Java, Indonesia; ^3^Microbiology Laboratory, Faculty of Medicine, Brawijaya University, Malang, East Java, Indonesia

## Abstract

A study was designed to investigate ameliorates effect of combined vitamins C and E able to against depot-medroxyprogesterone acetate- (DMPA-) induced ovarian oxidative stress in rat. Twenty-five female Wistar rats were divided into the following groups (*n* = 5 rats each): control (untreated) (C); depot-medroxyprogesterone acetate (DMPA); DMPA plus green vitamin C (at dose of 0.2 mg/gram; 0.4 mg/gram; 0.8 mg/gram) and vitamin E (0.04 IU/gram). The treatment with combined vitamins C and E was performed for four weeks. Analysis of malondialdehyde (MDA) level as a marker of oxidative stress was done colorimetrically. Analysis of SOD level was done by enzyme linked immunosorbent assay (ELISA) technically. This increase in ovarium MDA was significantly (*P* < 0.05) attenuated by medium dose treatments of combined vitamins C and E. DMPA insignificantly decreased SOD levels compared to the untreated group. This decrease in ovarian SOD level was significantly attenuated by all doses of the combined vitamins C and E. In conclusion, DMPA induces ovarian oxidative stress. Combined vitamins C and E prohibit the increase in ovarian lipid peroxidation, at least in part by modulating of superoxide dismutase. Therefore, this may provide an antioxidant therapy for attenuating the ovarian toxicity found in the DMPA therapy.

## 1. Introduction

Natural or synthetic xenobiotics, including drug, can decrease in the number of follicles, with a loss of ovarian cells (oogonia, oocytes, and somatic cells) [1]. One of this toxic effect due to trigger oxidative stress [2]. Depot-medroxyprogesterone acetate (DMPA) is an injectable form of progestin. Hormonal contraception is most often used by women in the United States and developing countries, including Indonesia. As a routine contraceptive use, the effectiveness of this compound is almost 90 days with the pregnancy rate of less than 1% [3–6]. The whole morphological alteration in the ovary after DMPA therapy appeared to be the atresia of the follicular apparatus with degeneration of the growing follicles [7].

Previous studies showed that DMPA trigger ovulation blockade marked by low levels of FSH and LH [8, 9]. Plasma estradiol is also significantly suppressed in the early follicular phase range and progesterone remains very low for months [9]. Estrogens, like vitamin E, which possess a phenolic hydroxyl group, also act as antioxidant and lipid peroxidation inhibitor in several models. Furthermore, these compounds are also able to inhibit the generation of peroxide radicals [10–12]. In certain conditions when this concentration is not equivalent to stressor, the oxidative stress will happen. In order to avoid oxidative stress, the supplementation with exogenous antioxidant should be performed.

Water-soluble antioxidants (vitamin C, phenolic compounds including flavonoids) and lipid-soluble antioxidants (vitamin E, carotenoids) are natural antioxidants that are hypothesized to have the potential to intervene in the development of atherosclerosis and cardiovascular disease by modulating redox sensitive steps in disease progression [13]. Combined supplementation of vitamin C and vitamin E is the best choice for antioxidant treatment. To the best of our knowledge, no study has evaluated the effect of DMPA on oxidative stress and antioxidant changes in ovarian tissue. Accordingly, we measured the level of MDA and superoxide dismutase in ovaries of rats that received DMPA. In addition, we also repair these changes by combining vitamins C and E.

## 2. Material and Methods

### 2.1. Animals

Twenty-five female Wistar rats, weighing 100–125 g, purchased from Physiology Laboratory were housed in an air-conditioned room at 25 ± 1°C and 65–70% relative humidity with a 12 h light-dark cycle. These animals were divided into the following groups (*n* = 5 rats each): control group (C); depot-medroxyprogesterone acetate (DMPA) group; and DMPA plus combined vitamins C and E group. The doses of vitamin C were 0.2, 0.4, and 0.8 mg/gram body weight per day. The dose of vitamin E was 0.04 IU/gram body weight per day. Previous studies found that mean values showed that plasma lipid standardised *α*-tocopherol increased with ascorbic acid supplementation [14]. The protocol used in this study was approved by the Ethic Committee for Animal Experimentation of the University of Brawijaya. Diets were made following the American Institute of Nutrition (AIN) recommendations. The animals were given food and water ad libitum during the experimental period.

### 2.2. DMPA Treatment

DMPA (Depo Progestin) injected every single week for four weeks at a dose of 2.7 mg/rat/week were diluted with 0.2 mL of saline and injected intramuscularly. DMPA dose is calculated according to the conversion of the human dose to rats.

### 2.3. Vitamins C and E

Vitamin C was dissolved with aquades 0.5 cc, but vitamin E was dissolved with sesame oil 0.5 cc. All these substances were orally treatment using gavage into rats at 10 a.m. every day for four weeks.

### 2.4. Tissue Sampling

At the end of the treatment, the animals in all groups were anesthetized. The ovary was collected, weighed, and later rinsed with physiological saline. All samples were stored at −80°C until analyzed.

### 2.5. Malondialdehyde Analysis

The BIOXYTECH MDA-586 Spectrophotometric Assay for Malondialdehyde assay kit (Catalog number 21044) was purchased from Oxis International, Inc. (Foster City, CA 94404 United States). The analysis was done according to detailed procedures in the kit.

### 2.6. Superoxide Dismutase Analysis

The EnzyChrom superoxide dismutase assay kit (Catalog number ESOD100) was purchased from BioAssay Systems (Hayward, CA, USA). The analysis was done according to detailed procedures in the kit.

### 2.7. Ethics

This research has been approved by the research ethics committee, Faculty of Medicine, University of Brawijaya, Malang, Indonesia.

### 2.8. Statistical Analysis

Data are presented as mean ± SD and differences between groups were analyzed using 1-way ANOVA with SPSS 15.0 statistical package. The post hoc test was used if the ANOVA was significant. *P* < 0.05 was considered statistically significant.

## 3. Results

Table 1 presents the ovarian weight from each experimental group. The ovarian weight was insignificantly lower in the DMPA group compared with the untreated group (*P* > 0.05). The lowest and highest dose of treatment significantly increased the ovarian weight compared with a DMPA group (*P* < 0.05). The lowest dose of treatment is able to reach a similar level with control group (*P* > 0.05).

Table 2 presents the ovarian MDA from each experimental group. The level of MDA was significantly higher in the DMPA group compared to the untreated control group (*P* < 0.05). Out of the 0.2 mg/gram, 0.4 mg/gram, and 0.8 mg/gram doses of vitamin C combined with vitamin E (0.04 IU/gram), only the middle dose significantly prevented DMPA-induced increase in MDA level in ovarian tissue. This dose achieved the MDA level similar to control group (*P* > 0.05).

Table 3 presents the SOD level in the ovary from each experimental group. The level of SOD was insignificantly reduced in the DMPA group compared to the untreated control group (*P* > 0.05). All doses of combined vitamins C and E significantly prevented DMPA-induced decrease in SOD level (*P* < 0.05), to reach the level in the control group (*P* > 0.05).

## 4. Discussion

In the current study, the ovarian weight was insignificantly lower in the DMPA group compared with the control (untreated) group (*P* > 0.05). Losing weight of ovaries may be due to the apoptosis effect of progestin of DMPA. Previous study shows that the monkeys treated with the progestin-component of the oral contraceptive (levonorgestrel) have increased apoptosis in the ovarian epithelium cells as compared with controls and ethinyl estradiol-treated monkeys [15]. The lowest and highest dose of treatment significantly increased the ovarian weight compared with a DMPA group (*P* < 0.05). The lowest dose of treatment is able to reach a similar level with control group (*P* > 0.05). This finding indicated that combined vitamins C and E treatment inhibits the apoptosis of ovarian tissue in the DMPA treatment group. Previous studies found that combined vitamins C and E act as antiapoptosis [16–20].

In this study, the level of MDA was significantly greater in the DMPA group compared to the untreated control group (*P* < 0.05). This finding indicates that DMPA increase lipid peroxidation of ovarian tissue. Previous studies showed that oral contraceptives containing 0.03 mg ethinylestradiol and 3 mg drospirenone significantly increase in the mean levels of lipid peroxides and oxidized LDLs [20]. Our data showed that the level of SOD tended to be reduced in the DMPA group compared to the untreated (control) group, although not significant (*P* > 0.05). We hypothesized that Habber-Weis reaction and downregulation of antioxidant are involved in this lipid peroxidation effect. The levels of Cu and Cu/Zn ratio were significantly increased in subjects who received oral contraceptives containing 0.03 mg ethinylestradiol and 3 mg drospirenone [21].

The medium dose of combined vitamins C and E significantly prevented DMPA-induced increase MDA level in ovarian tissue. This finding is consistent with and extended previous studies that supplementation with vitamins C and E significantly reduced plasma MDA levels in the oral contraceptive group by increasing the activity of GPx and GR [22]. Our finding showed that all doses of combined vitamins C and E significantly prevented DMPA-induced decrease in SOD level (*P* < 0.05) to reach the level in the control group (*P* > 0.05). Previous studies showed that, in continuous oxidative stress, there was an increase in the concentration of ascorbate radical that show as a peak followed by steady decline. After the virtual disappearance of the ascorbate radical, the appearance of the tocopheroxyl radical exists [23]. Besides, previous finding showed that mean values showed that plasma lipid standardised *α*-tocopherol increased with ascorbic acid supplementation [14].

In conclusion, DMPA induces ovarian oxidative stress. Combined vitamins C and E prohibit the increase in ovarian lipid peroxidation, at least in part by modulating of superoxide dismutase. Therefore, this may provide an antioxidant therapy for attenuating the ovarian toxicity found in the DMPA therapy.

## Figures and Tables

**Table 1:** The ovarian weight in each experimental group.

Level	Control	DMPA	DMPA + vitamin E (0.04 IU/gram body weight)		
			Vitamin C (0.2 mg)	Vitamin C (0.4 mg)	Vitamin C (0.8 mg)
Weight (gram)	72.40 ± 22.557	56.40 ± 15.192	102.6 ± 18.863^ab^	80.20 ± 15.547	92.60 ± 18.474^b^

Note: values are presented as mean ± SD; ^a^ *P* < 0.05; in comparison with control group; ^b^ *P* < 0.05; in comparison with DMPA group; DMPA: depot-medroxyprogesterone acetate B.

**Table 2:** Levels of ovarian malondialdehyde in each experimental group.

Level	Control	DMPA	DMPA + vitamin E (0.04 IU/gram body weight)		
			Vitamin C (0.2 mg)	Vitamin C (0.4 mg)	Vitamin C (0.8 mg)
MDA (*μ*M)	0.393 ± 0.053	1.339 ± 0.888^a^	0.933 ± 0.79	0.357 ± 0.116^b^	0.502 ± 0.086

Note: values are presented as mean ± SD; ^a^ *P* < 0.05; in comparison with control group; ^b^ *P* < 0.05; in comparison with DMPA group; MDA: malondialdehyde; DMPA: depot-medroxyprogesterone acetate B; *μ*M: mikromolar.

**Table 3:** Levels of ovarian superoxide dismutase in each experimental group.

Level	Control	DMPA	DMPA + vitamin E (0.04 IU/gram body weight)		
			Vitamin C (0.2 mg)	Vitamin C (0.4 mg)	Vitamin C (0.8 mg)
SOD (*μ*/mL)	1546.667 ± 232.504	1176 ± 502.536	2741 ± 2221.203^b^	2366.25 ± 378.756^b^	2147 ± 449.619^b^

Note: values are presented as mean ± SD; ^b^ *P* < 0.05; in comparison with DMPA group; MDA: malondialdehyde; DMPA: depot-medroxyprogesterone acetate B; *μ*/mL: mikron/milliliter.

## References

[1] Regan K. S., Cline J. M., Creasy D. (2005). STP position paper: ovarian follicular counting in the assessment of rodent reproductive toxicity. *Toxicologic Pathology*.

[2] Mingels M. J. J. M., Geels Y. P., Pijnenborg J. M. A. (2013). Histopathologic assessment of the entire endometrium in asymptomatic women. *Human Pathology*.

[3] Erkkola R., Landgren B.-M. (2005). Role of progestins in contraception. *Acta Obstetricia et Gynecologica Scandinavica*.

[4] Kaunitz A. M. (2000). Injectable contraception: new and existing options. *Obstetrics and Gynecology Clinics of North America*.

[5] Jain J., Dutton C., Nicosia A., Wajszczuk C., Bode F. R., Mishell D. R. (2004). Pharmacokinetics, ovulation suppression and return to ovulation following a lower dose subcutaneous formulation of Depo-Provera®. *Contraception*.

[6] Cheng Toh Y., Jain J., Rahnny M. H., Bode F. R., Ross D. (2004). Suppression of ovulation by a new subcutaneous depot medroxyprogesterone acetate (104 mg/0.65 ml) contraceptive formulation in asian women. *Clinical Therapeutics*.

[7] Bhowmik T., Mukherjea M. (1988). Histological changes in the ovary and uterus of rat after injectable contraceptive therapy. *Contraception*.

[8] Mishell D. R., Kletzky O. A., Brenner P. F., Roy S., Nicoloff J. (1977). The effect of contraceptive steroids on hypothalamic-pituitary function. *American Journal of Obstetrics and Gynecology*.

[9] Jeppson S., Gershagen S., Johansson E. D. B. (1982). Plasma levels of medroxyprogesterone acetate (MPA), sex hormone binding globulin, gonadal steroids, gonadotrophins and prolactin in women during long-term use of depo-MPA (Depo-Provera) as a contraceptive agent. *Acta Endocrinologica*.

[10] Ulas M., Cay M. (2010). The effects of 17*β*-estradiol and vitamin E treatments on oxidative stress and antioxidant levels in brain cortex of diabetic ovariectomized rats. *Acta Physiologica Hungarica*.

[11] Ulas M., Cay M. (2009). Effects of vitamin E and 17-*β* estradiol on plasma lipid and lipid peroxidation levels in ovariectomized and diabetic rats. *Fırat University Veterinary Journal of Health Sciences*.

[12] Bureau I., Gueux E., Mazur A., Rock E., Roussel A.-M., Rayssiguier Y. (2003). Female rats are protected against oxidative stress during copper deficiency. *Journal of the American College of Nutrition*.

[13] Kinsella J. E., Frankel E. N., German J. B. (1993). Possible mechanism for the protective role of antioxidants in wine and plant foods. *Food Technology*.

[14] Hamilton I. M. J., Gilmore W. S., Benzie I. F. F., Mulholland C. W., Strain J. J. (2000). Interactions between vitamins C and E in human subjects. *British Journal of Nutrition*.

[15] Rodriguez G. C., Walmer D. K., Cline M. (1998). Effect of progestin on the ovarian epithelium of macaques: cancer prevention through apoptosis?. *Journal of the Society for Gynecologic Investigation*.

[16] Tannetta D. S., Sargent I. L., Linton E. A., Redman C. W. G. (2008). Vitamins C and E inhibit apoptosis of cultured human term placenta trophoblast. *Placenta*.

[17] Guney M., Oral B., Take G., Giray S. G., Mungan T. (2007). Effect of fluoride intoxication on endometrial apoptosis and lipid peroxidation in rats: role of vitamins E and C. *Toxicology*.

[18] Güney M., Oral B., Demirin H. (2007). Evaluation of caspase-dependent apoptosis during methyl parathion-induced endometrial damage in rats: ameliorating effect of Vitamins E and C. *Environmental Toxicology and Pharmacology*.

[19] Oral B., Guney M., Demirin H. (2006). Endometrial damage and apoptosis in rats induced by dichlorvos and ameliorating effect of antioxidant Vitamins E and C. *Reproductive Toxicology*.

[20] Traber M. G., Stevens J. F. (2011). Vitamins C and E: beneficial effects from a mechanistic perspective. *Free Radical Biology and Medicine*.

[21] De Groote D., d'Hauterive S. P., Pintiaux A. (2009). Effects of oral contraception with ethinylestradiol and drospirenone on oxidative stress in women 18–35 years old. *Contraception*.

[22] Zal F., Mostafavi-Pour Z., Amini F., Heidari A. (2012). Effect of vitamin E and C supplements on lipid peroxidation and GSH-dependent antioxidant enzyme status in the blood of women consuming oral contraceptives. *Contraception*.

[23] Sharma M. K., Buettner G. R. (1993). Interaction of vitamin C and vitamin E during free radical stress in plasma: an ESR study. *Free Radical Biology and Medicine*.

